# Associations between multiple inflammatory biomarkers and the risk of developing kidney stones

**DOI:** 10.1186/s12894-025-01735-5

**Published:** 2025-03-12

**Authors:** Jun Ho Lee, Hoyoung Bae

**Affiliations:** 1 Department of Urology, Nowon Eulji Medical Center, Eulji University, Seoul, South Korea; 2 Department of Urology, Boramae Medical Center, Seoul National University, Seoul, South Korea

**Keywords:** Nephrolithiasis, Inflammatory marker, NLR, LMR, SII

## Abstract

**Objectives:**

Nephrolithiasis, with a prevalence of 9% and increasing worldwide, has a recurrence rate close to 50%. Urinary stones significantly impact quality of life and impose substantial economic burdens on patients and healthcare systems. Systemic inflammation is postulated as a risk factor for urinary stones. Previous studies have identified associations between inflammatory markers and kidney stones, but these often rely on patient recall, introducing potential recall bias. This study investigates whether inflammatory markers vary according to the presence of nephrolithiasis using health check-up data from a large cohort in South Korea.

**Materials and methods:**

Data were collected from participants in health checkups at a university hospital in Seoul between 2010 and 2020. The study included 18,243 males and 12,919 females who underwent blood tests, KUB (Kidneys, Ureters, and Bladder) radiography, and ultrasound examinations. Only stones larger than 5 mm were counted, enrolling 328 males and 99 females with kidney stones. Exclusion criteria included pyuria, congenital renal deformities, renal cancer, kidney transplant, and diuretic use. Inflammatory markers assessed included the neutrophil-to-lymphocyte ratio (NLR), lymphocyte-to-monocyte ratio (LMR), and systemic immune-inflammatory index (SII). The primary outcome was the presence of nephrolithiasis, detected using combined ultrasonography and KUB radiography. Logistic regression analyses determined the association between inflammatory markers and nephrolithiasis, adjusting for confounders such as age, BMI, blood pressure, triglycerides, LDL, HDL, creatinine, BUN, uric acid, fasting glucose, calcium, and medical history.

**Results:**

In females, an LMR ≤ 5.02 (OR: 2.30, 95% CI: 1.47–3.61, *p* < 0.001), NLR > 1.94 (OR: 1.97, 95% CI: 1.24–3.12, *p* = 0.004), and SII > 484.05 (OR: 2.12, 95% CI: 1.38–3.24, *p* < 0.001) were significantly associated with kidney stones after adjusting for confounders. In males, an LMR ≤ 7.79 (OR: 1.82, 95% CI: 1.33–2.49, *p* < 0.001) and NLR > 1.32 (OR: 1.55, 95% CI: 1.12–2.15, *p* = 0.009) were significantly associated with kidney stones, but SII > 560.11 was not (OR: 1.21, 95% CI: 0.87–1.68, *p* = 0.255), after adjusting. The significant relationships between these inflammatory markers and kidney stones were maintained only in participants aged ≥ 50 years. Specifically, in females aged ≥ 50 years, LMR ≤ 5.02 (OR: 2.38, 95% CI: 1.52–3.74, *p* < 0.001), NLR > 1.94 (OR: 2.05, 95% CI: 1.30–3.24, *p* = 0.002), and SII > 484.05 (OR: 2.18, 95% CI: 1.43–3.32, *p* < 0.001) were significant predictors of nephrolithiasis. In males aged ≥ 50 years, LMR ≤ 7.79 (OR: 1.90, 95% CI: 1.38–2.62, *p* < 0.001) and NLR > 1.32 (OR: 1.62, 95% CI: 1.17–2.25, *p* = 0.004) were significant predictors.

**Conclusion:**

Elevated inflammatory markers are significantly associated with the presence of kidney stones, particularly in individuals aged 50 years or older. These findings suggest that systemic inflammation plays a crucial role in the pathogenesis of nephrolithiasis, especially in the older population. The results imply that inflammation contributes to the increasing prevalence of urinary stones with age, highlighting the importance of managing systemic inflammation in preventing nephrolithiasis. Future research would be needed to explore causal relationships and investigate whether anti-inflammatory interventions can reduce the risk of kidney stones.

**Supplementary Information:**

The online version contains supplementary material available at 10.1186/s12894-025-01735-5.

## Introduction

The prevalence of nephrolithiasis is 9% in the US [1] and is increasing worldwide, and the nephrolithiasis recurrence rate is close to 50% [2]. Urinary stones have a negative impact on quality of life and impose a significant economic burden on patients and health care systems. However, the pathogenesis of urinary stones is not clear. Considering its high prevalence, high recurrence rate, and economic burden, it is important to understand the underlying pathophysiology to help prevent urinary stone disease.

Systemic inflammation has been postulated to be a risk factor for urinary stones. The role of macrophagerelated inflammation in the formation of renal calcium oxalate crystals has been suggested in a previous laboratory study [3]. Clinical studies utilizing data from the National Health and Nutrition Examination Survey (NHANES) have revealed a significant association between inflammatory markers and the presence of kidney stones. Notably, elevated levels of C-reactive protein (CRP) [4] and a high neutrophil-to-lymphocyte ratio (NLR) have been correlated with an increased incidence of kidney stones [5]. Recently, a high systemic immune-inflammatory index (SII), which is a relatively new inflammatory marker, was also related to the risk of developing kidney stones in participants in their 20–40 s in a study using data from the NHANES 2007–2018 [6]. However, these studies relied on patient recall for information regarding kidney stones, potentially diminishing the reliability of findings due to recall bias. Additionally, big data other than those from the NHANES are still scarce. Moreover, data on the relationship between the risk of developing urinary stones and inflammatory markers other than CRP are scarce. Therefore, we conducted this study to address some of these limitations and to investigate whether inflammatory markers vary according to the presence of nephrolithiasis using health check-up data from 31,162 participants.

## Materials and methods

### Subjects

Data on men who participated in health checkups at one university hospital in Seoul, South Korea, during the period from 2010 to 2020 were collected. Participants voluntarily underwent health checkups at the health screening centre.

Anthropometric measurements (height and weight), blood pressure measurements, basic blood chemistry, serum uric acid, and lipid profile analyses were included in the basic health screening. Plain-film kidney, ureter and bladder radiography (KUB) and abdominal ultrasonography were performed. Serum was drawn between 7:00 and 9:00 AM after overnight fasting. Medical histories were collected by trained nurses.

In this study, we included men (aged ≥ 20 years) who simultaneously underwent blood, KUB test, and ultrasound examination on the day of the basic health checkup. The exclusion criteria were as follows: had pyuria and recurrent urinary tract infection (*n* = 67); had any congenital renal deformities, polycystic kidney disease, dysgenesis or hypoplasia, renal cancer or renal tumour, or a kidney transplant (*n* = 17); and were taking diuretics (*n* = 98). Finally, 18,243 males and 12,919 females were enrolled.

To evaluate the associations between inflammatory markers and the risk of developing kidney stones, age; body mass index (BMI); blood pressure; concentrations of triglyceride (TG), low-density lipoprotein (LDL) cholesterol, high-density lipoprotein (HDL) cholesterol, creatinine (Cr), blood urea nitrogen (BUN), uric acid, fasting glucose, and calcium; and medical history data were examined. This study was conducted in accordance with the Declaration of Helsinki for experiments involving humans. The Institutional Review Board reviewed and approved the study protocol (approval number 2024-06-013).

### Kidney stones

For stone detection, We used combined ultrasonography with KUB radiography. A comprehensive review of the literature showed that combining KUB with ultrasound improves the sensitivity compared to that of ultrasound alone, with estimates of sensitivity and specificity ranging from 58 to 100% and 37–100%, respectively [7].

KUB was taken immediately prior to ultrasonography, and investigators reviewed the KUB before performing ultrasonography. Nephrolithiasis was suspected when calcifications were identified along the anatomic course of the kidney on the KUB. The KUB was used to guide the radiologist to the area of suspected urinary calculi.

All of the ultrasonography examinations were performed by attending radiologists using an iU22 system (Philips Medical Systems, Amsterdam, Netherlands). B-mode ultrasonography was used to physically differentiate between stones and surrounding tissues to detect the stones. Bright echogenic structures with a nonechogenic shadow in the ultrasonographic image were considered to indicate nephrolithiasis. Small calculi (< 3 mm) might not show acoustic shadowing. However, we did not define bright echogenic structures without a nonechogenic shadow as nephrolithiasis to include more clinically important renal stones [8] because it has been reported that larger asymptomatic renal stones (> 5 mm) are more likely to become symptomatic in long-term follow-up studies [9]. Therefore, we only included stones larger than 5 mm that presented with an acoustic shadow on ultrasonography.

### Confounders

Age [10], hypertension [10], BMI [10], dyslipidaemia [11], renal function [12], fasting glucose concentrations (diabetes) [13], serum calcium concentrations [14], and serum uric acid levels [15] were associated with urinary stones in previous studies. Therefore, we adjusted for the aforementioned factors to elucidate the associations between systemic inflammatory markers and the risk of developing nephrolithiasis.

### Systemic inflammation assessment

Complete blood cell counts with differential counts were assessed using automated analysers (fluorescence flow cytometry, electrical impedance, Sysmex 2100). The NLR (absolute neutrophil count/absolute lymphocyte count), lymphocyte-to-monocyte ratio (LMR; absolute lymphocyte count/absolute monocyte count), and SII (absolute platelet count x absolute neutrophil count/absolute lymphocyte count) were calculated to assess systemic inflammation.

### Statistical analysis

The primary aim of our study was to elucidate the relationships between inflammatory markers and the risk of developing nephrolithiasis. The demographics were assessed using descriptive statistics. The cut-off points for the NLR, LMR, and SII, which are indicators of the presence of kidney stones, were determined using receiver operating characteristic (ROC) curves, which enabled the calculation of the total area under the ROC curve and cut-off points with better sensitivity and specificity.

We used binary logistic regression to determine the associations between the cut-off values of the NLR, LMR, and SII for the risk of developing nephrolithiasis before and after adjusting for confounders. Odds ratios (ORs) with 95% confidence intervals (CIs) were calculated before and after adjusting for all confounders. Additionally, after stratification of the participants according to the age of 50 years, the ORs of the cut-off values for the NLR, LMR, and SII for the risk of developing nephrolithiasis were also calculated.

All tests were two-sided, with statistical significance set at *P* < 0.05. Data analysis was conducted using the R statistical software (version 2.13.1, R Foundation for Statistical Computing, Vienna, Austria).

## Results

A total of 328 males and 99 females had kidney stones. The demographic data of the study participants are given in Table 1.

**Table 1:** Participant characteristics

Variable	Female	Male
	(*N* = 12919)	(*N* = 18243)
Age (y)	47.0 [39.0;55.0]	48.0 [41.0;55.0]
BMI (kg/m^2^)	22.3 [20.5;24.6]	24.8 [23.1;26.9]
SBP (mmHg)	116.0 [107.0;127.0]	124.0 [116.0;132.0]
DBP (mmHg)	70.0 [64.0;77.0]	75.0 [69.0;82.0]
TG (mg/dL)	84.0 [61.0;121.0]	126.0 [87.0;182.0]
LDL (mg/dL)	115.0 [94.0;138.0]	123.0 [100.0;146.0]
HDL (mg/dL)	59.0 [50.0;69.0]	47.0 [41.0;55.0]
Cr (mg/dL)	0.8 [ 0.7; 0.8]	1.0 [ 0.9; 1.1]
BUN (mg/dL)	12.3 [10.3;14.7]	13.8 [11.8;16.2]
Fasting glucose (mg/dL)	86.0 [81.0;94.0]	91.0 [84.0;101.0]
Uric acid (mg/dL)	4.4 [ 3.8; 5.1]	6.0 [ 5.2; 6.9]
Calcium (mg/dL)	9.2 [ 9.0; 9.5]	9.3 [ 9.1; 9.6]
Platelets (×10^9^/L)	252 [ 217; 292]	241.00 [ 209; 277]
Neutrophils (×10^9^/L)	2.78 [2.18;3.52]	3.02 [2.44;3.78]
Lymphocytes (×10^9^/L)	1.59 [1.33;1.91]	1.77 [1.48;2.11]
Monocytes (×10^9^/L)	0.24 [ 0.20; 0.30]	0.31 [0.25; 0.37]
NLR	1.7 [ 1.3; 2.2]	1.7 [ 1.4; 2.2]
LMR	6.6 [ 5.3; 8.2]	5.8 [ 4.7; 7.1]
SII	436.4 [320.7;593.8]	411.4 [309.5;550.1]
Kidney stones		
No	12,820 (99.2%)	17,915 (98.2%)
Yes	99 (0.8%)	328 (1.8%)

Values are presented as the median [interquartile range] or number (percentage)

BMI = body mass index; SBP = systolic blood pressure; DBP = diastolic blood pressure; TG = triglyceride; LDL = low-density lipoprotein cholesterol; HDL = high-density lipoprotein cholesterol; Cr = creatinine; BUN = blood urea nitrogen; NLR = high neutrophil-to-lymphocyte ratio; LMR = lymphocyte-to-monocyte ratio; SII = systemic immune-inflammatory index

Table 2 lists the area under the ROC curve, significance levels (*p* values), cut-off values, sensitivity and specificity for the LMR, NLR, and SII as discriminators of the presence of kidney stones. In females, the cut-off values for the LMR, NLR, and SII were 5.02, 1.94, and 484.05, respectively. In males, the cut-off values for the LMR, NLR, and SII were 7.79, 1.32, and 560.11, respectively.

**Table 2:** Receiver operating characteristic (ROC) curve analysis of inflammatory biomarkers as discriminators of the presence of kidney stones

Sex	Variable	AUC_ROC_ curve (95% CI)	*p*	Cut-off point	Sensitivity (%)	Specificity (%)
Female	LMR	0.563 (0.555 to 0.572)	0.0297	≤ 5.02	31.31	80.72
	NLR	0.572 (0.564 to 0.581)	0.0085	> 1.94	52.53	61.69
	SII	0.555 (0.546 to 0.564)	0.0417	> 484.05	52.53	59.07
Male	LMR	0.523 (0.516 to 0.530)	0.1352	≤ 7.79	88.96	16.56
	NLR	0.527 (0.519 to 0.534)	0.0816	> 1.32	84.97	22.78
	SII	0.5 (0.493 to 0.508)	0.9786	> 560.11	19.02	76.29

AUC_ROC_=area under the ROC curve; CI = confidence interval, LMR = lymphocyte–monocyte ratio, NLR = neutrophil–lymphocyte ratio, SII = systemic immune–inflammation index

In females, an LMR ≤ 5.02, an NLR > 1.94, and an SII > 484.05 were significantly related to the risk of developing kidney stones before and after adjustment for confounders (Table 3). In males, an LMR ≤ 7.79 and an NLR > 1.32 were significantly related to the risk of developing kidney stones before and after adjusting for confounders. However, an SII > 560.11 was not significantly related to the risk of developing kidney stones.

**Table 3:** Inflammatory biomarkers and risk of nephrolithiasis

Female						Male				
Variable			Model 1, OR (95% CI), p	Model 2, OR (95% CI), p		Variable (mg/dL)			Model 1, OR (95% CI), p	Model 2, OR (95% CI), p
LMR	> 5.02		Reference	Reference		LMR	> 7.79		Reference	Reference
	≤ 5.02		1.908 (1.245–2.925), 0.003	1.672 (1.050–2.664), 0.001			≤ 7.79		1.598 (1.128–2.265), 0.008	1.445 (1.007–2.074), 0.046
NLR	≤ 1.94		Reference	Reference		NLR	≤ 1.32		Reference	Reference
	> 1.94		1.781 (1.198–2.646), 0.006	1.838 (1.202–2.808), 0.005			> 1.32		1.666 (1.227–2.262), 0.001	1.638 (1.181–2.273), 0.003
SII	≤ 484.05		Reference	Reference		SII	≤ 560.11		Reference	Reference
	> 484.05		1.596 (1.074–2.372), 0.021	1.685 (1.101–2.578), 0.016			> 560.11		0.771 (0.584-1.017), 0.065	0.776 (0.580-1.038), 0.776

Model 1: Unadjusted model

Model 2: age, BMI, SBP, DBP, TG, LDL, HDL, fasting glucose, BUN, Cr, uric acid, and calcium

OR = odds ratio, CI = confidence interval, BMI = body mass index; SBP = systolic blood pressure; DBP = diastolic blood pressure; TG = triglyceride; LDL = low-density lipoprotein cholesterol; HDL = high-density lipoprotein cholesterol; Cr = creatinine; BUN = blood urea nitrogen; hs-CRP = high-sensitivity C-reactive protein

The significant relationships between several inflammatory markers (the LMR, NLR, and SII in females and the LMR and NLR in males) and the risk of developing kidney stones were maintained only in those aged ≥ 50 years but not in those aged < 50 years after stratification of participants according to age (Table 4).

**Table 4:** Subgroup analysis of the associations between inflammatory biomarkers and the risk of developing kidney stones

Sex	Stratified by age (years)	Variable		Model 1, OR (95% CI), *p*		Model 2, OR (95% CI), *p*
Male	< 50	LMR > 7.79		Reference		Reference
		LMR ≤ 7.79		1.295 (0.786-2.135), 0.310		1.225 (0.729–2.059), 0.443
	≥ 50	LMR > 7.79		Reference		Reference
		LMR ≤ 7.79		1.868 (1.146–3.045), 0.012		1.719 (1.035–2.854), 0.036
	< 50	NLR ≤ 1.32		Reference		Reference
		NLR > 1.32		1.209 (0.785-1.861), 0.389		1.347 (0.843-2.151), 0.213
	≥ 50	NLR ≤ 1.32		Reference		Reference
		NLR > 1.32		2.176 (1.404–3.374), 0.001		2.039 (1.284–3.236), 0.003
	< 50	SII ≤ 560.11		Reference		Reference
		SII > 560.11		0.849 (0.561-1.286), 0.440		0.864 (0.556-1.343), 0.864
	≥ 50	SII ≤ 560.11		Reference		Reference
		SII > 560.11		0.742 (0.510-1.078), 0.117		0.720 (0.456-1.066), 0.100
Female	< 50	LMR > 5.02		Reference		Reference
		LMR ≤ 5.02		1.559 (0.809-3.005), 0.185		1.199 (0.575-2.502), 628
	≥ 50	LMR > 5.02		Reference		Reference
		LMR ≤ 5.02		2.672 (1.520–4.695), 0.001		2.283 (1.242–4.196), 0.008
	< 50	NLR ≤ 1.94		Reference		Reference
		NLR > 1.94		1.131 (0.617-2.077), 0.690		1.062 (0.553 − 2.040), 0.857
	≥ 50	NLR ≤ 1.94		Reference		Reference
		NLR > 1.94		3.129 (1.848–5.298), < 0.001		2.741 (1.584–4.802), < 0.001
	< 50	SII ≤ 484.05		Reference		Reference
		SII > 484.05		1.200 (0.654-2.203), 0.555		1.206 (0.624-2.331), 0.577
	≥ 50	SII ≤ 484.05		Reference		Reference
		SII > 484.05		2.512 (1.488–4.239), 0.001		2.183 (1.250–3.812), 0.006

Model 1: Unadjusted model

Model 2: age, BMI, SBP, DBP, fasting glucose, BUN/Cr, uric acid, hs-CRP, and calcium

OR = odds ratio, BMI = body mass index; SBP = systolic blood pressure; DBP = diastolic blood pressure; HDL = high-density lipoprotein cholesterol; Cr = creatinine; BUN = blood urea nitrogen; hs-CRP = high-sensitivity C-reactive protein

## Discussion

The objective of this study is to ascertain the correlation between the presence of kidney stones at the time of the study and inflammation markers. Therefore, inclusion criteria encompass individuals both with and without a history of stone formation.

In this study, an LMR ≤ 5.02, an NLR > 1.94, and an SII > 484.05 were significantly and independently related to the risk of developing kidney stones in females. An LMR ≤ 7.79 and an NLR > 1.32 were also significantly and independently related to the risk of developing kidney stones in males. The significant relationships between several inflammatory markers (the LMR, NLR, and SII in females and the LMR and NLR in males) and the risk of developing kidney stones remained only in those aged 50 years or older after stratification by age group.

To comprehend the implications of inflammatory markers, it is essential to understand their basic functions. During immune responses, neutrophils are the first cells to reach infection sites and exhibit potent bactericidal activities. Their involvement extends to cell killing during graft rejection and indicates nonspecific inflammation in various conditions, including atherosclerosis, autoimmune diseases, and cancer [16]. Lymphocytes play crucial roles in cytotoxic cell death and in inhibiting tumour cell proliferation and migration, reflecting specific immunity aimed at preventing new infections and combating tumour cells, thus representing host immune competence [17]. The NLR, calculated by dividing the neutrophil count by the lymphocyte count, has been utilized to indicate the immune-inflammatory activities of neutrophils and lymphocytes in various clinical settings, such as cancer progression, cardiovascular diseases, hypertension and kidney diseases [18, 19].

Monocytes, as precursors to macrophages, are pivotal in innate immune responses and their count is closely correlated with the risk of developing many different inflammatory diseases. The LMR, calculated by dividing the absolute lymphocyte count by the absolute monocyte count, is emerging as a significant systemic inflammatory marker. A decreased LMR has been associated with poor prognosis in various cancers [20].

Platelets, traditionally known for their role in haemostasis, also play crucial roles in pathogen defence and the progression of autoimmune disease, underscoring their importance as a component of the innate immune system. The SII integrates platelet counts and the NLR to simultaneously assess patients’ inflammatory and immune statuses. The SII has been identified as a prognostic marker in conditions such as cancer, intracerebral haemorrhage, and coronary stenosis [21].

Several studies have been conducted to investigate the associations between the risk of developing urinary stones and inflammatory markers, including CRP concentrations, the NLR, the MLR, and the SII. A study employing data from the NHANES 2007–2009 revealed a significant association between elevated CRP levels (3rd to 5th quintile) and the prevalence of kidney stones in individuals aged 20–39 years [22]. Another study (NHANES 2007–2014) reported a significant association between CRP levels and lifetime kidney stone prevalence across all age groups [23].

A study based on NHANES data from 2007 to 2014 revealed that a neutrophil-to-lymphocyte ratio (NLR) greater than 1.72 was associated with an increased prevalence of kidney stones and a greater number of stones passed, indicating the relevance of inflammatory markers other than CRP in this condition. However, the monocyte-to-lymphocyte ratio and the SII are not related to the risk of developing kidney stones [23]. Another study involving 22,220 NHANES participants between 2007 and 2018 revealed an independent association between the SII and the risk of developing kidney stones in individuals under 50 years old [6]. These studies presented slightly conflicting results compared to our research, which revealed significant relationships between the risk of developing kidney stones and the LMR, NLR, and SII in females aged ≥ 50 years and the LMR and NLR in males aged ≥ 50 years. A key methodological difference in our study is the use of current kidney stone status assessed via KUB and ultrasonography, as opposed to the reliance on self-reported lifetime prevalence of kidney stones in the aforementioned studies. The accuracy of patient recall is frequently compromised by imperfections such as random errors and recall bias. Additionally, it is known that recall ability can diminish with age [24]. These factors may have led to different results from those of previous studies. Nevertheless, the results of both previous NHANES studies and the current study, for which we used a large dataset, support the fact that systemic inflammation is significantly correlated with the risk of developing urinary stones.

The role of inflammation in urinary stone formation may be explained by the findings of previous laboratory studies. Crystals, which are precursors to renal stones, have been observed adhering to the luminal surfaces of cells that express hyaluronan, osteopontin, and CD44 [25]. This adherence increases the expression of inflammation-related genes within renal tubular cells. This genetic upregulation promotes the migration and phagocytosis of monocyte-macrophages [26]. The secretion of reactive oxygen species, chemokines, proinflammatory cytokines, and fibrotic factors is increased in macrophages that encounter calcium oxalate crystals. This response intensifies renal interstitial inflammation, which is a key feature of kidney stone disease. Furthermore, these macrophages enhance the recruitment of diverse immune cells—including monocytes, macrophages, neutrophils, dendritic cells, and T cells—to sites of inflammation [27]. In particular, M1 macrophages are often found in regions of Randall’s plaque formation, where they contribute to subepithelial mineralized tissue deposition at the papillary tip. Randall’s plaque itself is a critical site for stone development and is characterized by its unique fibrillar collagen composition [28].

Interestingly, in the present study, the associations between elevated inflammatory markers and the risk of developing kidney stones were observed exclusively in the group over 50 years of age. These results imply that inflammation is more important in the pathogenesis of stone formation in older patients than in younger patients and that inflammation may contribute to the increase in the prevalence of urinary stones with age. Ageing is marked by a condition known as ‘inflammaging’, which refers to chronic, sterile, low-grade inflammation characterized by continuous immune activation. This state contributes to the pathogenesis of various age-related diseases [29]. In our study, only participants older than 50 years showed significant correlations between kidney stones and inflammatory markers such as the LMR and NLR, likely reflecting these age-related immunological changes. The ageing immune response, characterized by a directional shift towards chronic inflammation, results in increased monocyte counts and decreased lymphocyte counts, underlying the observed trends. However, given that these are generally associated with ageing, further research is needed to explore the specific inflammatory responses related to the risk of developing kidney stones.

Another interesting finding was that the SII was associated with the risk of developing kidney stones in females but not in males. The differing results by sex raise the possibility that hormonal factors such as testosterone or estrogen may influence the relationship between the SII and kidney stone risk. While the precise mechanisms remain unclear, existing research suggests that immune responses can vary significantly between males and females. This underscores the need for further fundamental research to investigate these mechanisms in greater depth. A better understanding of these sex-specific differences in immune response could provide valuable insights for developing tailored therapeutic strategies for various conditions.

The prevalence of kidney stones in this study (1.37%) may appear low, as stones without an acoustic shadow on ultrasonography or those smaller than 5 mm were excluded. Additionally, some stones undetectable by KUB or ultrasound due to their composition were not accounted for, being visible only on CT.

### Limitation

A limitation of our study is that we used data from a single clinical health examination centre in a single region. Additionally, the cross-sectional nature of our study poses challenges in establishing clear causal relationships. To better understand the underlying causative factors, further longitudinal follow-up studies are warranted. We aim to address this limitation in future research to provide more definitive insights.

Another limitation of our study is that the cohort consisted of health check-up participants, which made it impossible to collect kidney stone samples. As a result, we were unable to determine the precise compositional details of the kidney stone. To address this limitation, future studies should focus on cohorts where samples can be obtained through endoscopic procedures or other appropriate methods.

However, we identified nephrolithiasis using an imaging study with a large cohort, and we adjusted for multiple confounders. Therefore, we believe that our data are represent the most robust recent evidence from studies that attempted to elucidate the relationship between inflammatory markers and the risk of developing nephrolithiasis.

## Conclusion

In our study, we revealed that the prevalence of kidney stones significantly increased when the serum inflammatory marker level was high. We believe that further follow-up studies of our findings are warranted to determine whether systemic inflammation can cause urinary stones in long-term prospective cohort studies or whether anti-inflammatory drugs could reduce kidney stone risk in prospective observational studies or clinical trials.

## Electronic supplementary material

Below is the link to the electronic supplementary material.

Supplementary Material 1

Supplementary Material 2

Supplementary Material 3

Supplementary Material 4

Supplementary Material 5

Supplementary Material 6

## Data Availability

This study is a retrospective analysis of anonymized health examination data collected from 2010 to 2020. Only the statistical data derived from these examinations are available. The analyzed data are stored in a secure database and can be accessed upon reasonable request to the corresponding author. Raw data are not available to maintain patient confidentiality.
