# Supplementary figures and images for: Associations between multiple inflammatory biomarkers and the risk of developing kidney stones
Source: BMC Urol. 2025 Mar 12;25:48. doi: 10.1186/s12894-025-01735-5 (PMC11900638; doi:10.1186/s12894-025-01735-5)

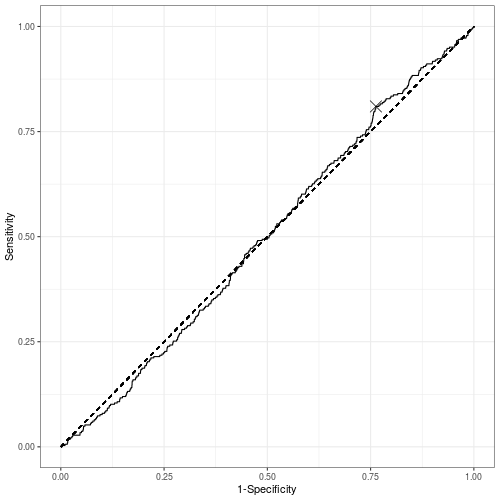

Supplement: Supplementary file 1 — Supplementary Material 1 [file 12894_2025_1735_MOESM1_ESM.png]

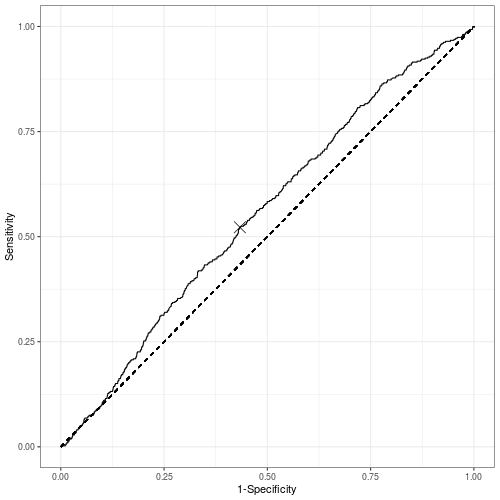

Supplement: Supplementary file 2 — Supplementary Material 2 [file 12894_2025_1735_MOESM2_ESM.png]

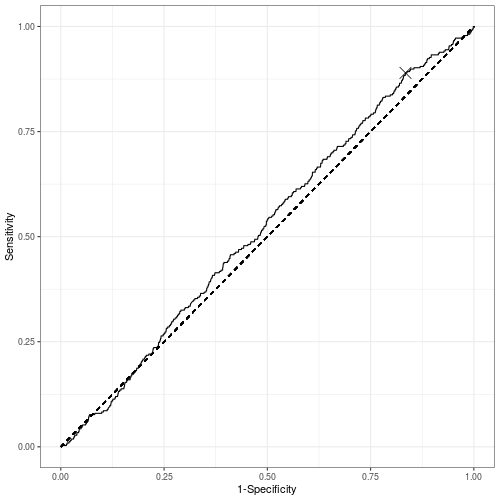

Supplement: Supplementary file 3 — Supplementary Material 3 [file 12894_2025_1735_MOESM3_ESM.png]

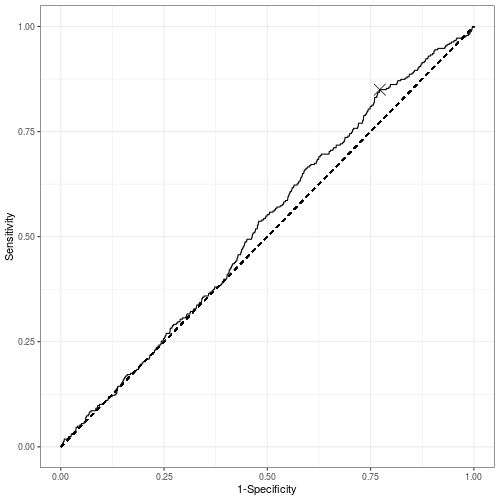

Supplement: Supplementary file 4 — Supplementary Material 4 [file 12894_2025_1735_MOESM4_ESM.png]

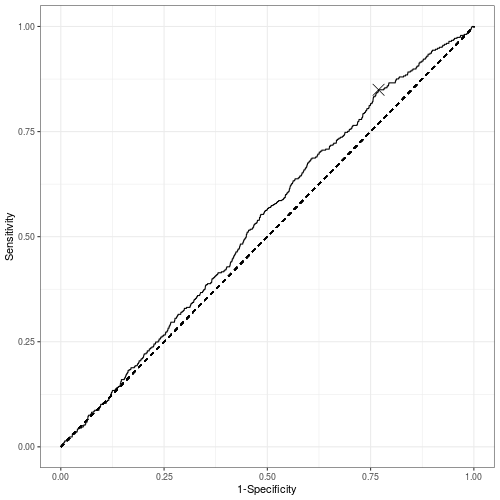

Supplement: Supplementary file 5 — Supplementary Material 5 [file 12894_2025_1735_MOESM5_ESM.png]
